# Sulfonamide inhibition studies of two β-carbonic anhydrases from the ascomycete fungus *Sordaria macrospora,* CAS1 and CAS2

**DOI:** 10.1080/14756366.2018.1425687

**Published:** 2018-01-24

**Authors:** Daniela Vullo, Ronny Lehneck, Stefanie Pöggeler, Claudiu T. Supuran

**Affiliations:** a Polo Scientifico, Laboratorio di Chimica Bioinorganica, Università degli Studi di Firenze, Sesto Fiorentino, Florence, Italy;; b Institute of Microbiology and Genetics, Department of Genetics of Eukaryotic Microorganisms, Georg-August-University Göttingen, Göttingen, Germany;; c Neurfarba Department, Sezione di Scienze Farmaceutiche e Nutraceutiche, Università degli Studi di Firenze, Sesto Fiorentino, Florence, Italy

**Keywords:** Carbonic anhydrase, fungus, *Sordaria macrospora*, sulfonamide, sulfamate, inhibitor

## Abstract

The two β-carbonic anhydrases (CAs, EC 4.2.1.1) recently cloned and purified from the ascomycete fungus *Sordaria macrospora*, CAS1 and CAS2, were investigated for their inhibition with a panel of 39 aromatic, heterocyclic, and aliphatic sulfonamides and one sulfamate, many of which are clinically used agents. CAS1 was efficiently inhibited by tosylamide, 3-fluorosulfanilamide, and 3-chlorosulfanilamide (*K*_I_s in the range of 43.2–79.6 nM), whereas acetazolamide, methazolamide, topiramate, ethoxzolamide, dorzolamide, and brinzolamide were medium potency inhibitors (*K*_I_s in the range of 360–445 nM). CAS2 was less sensitive to sulfonamide inhibitors. The best CAS2 inhibitors were 5-amino-1,3,4-thiadiazole-2-sulfonamide (the deacetylated acetazolamide precursor) and 4-hydroxymethyl-benzenesulfonamide, with *K*_I_s in the range of 48.1–92.5 nM. Acetazolamide, dorzolamide, ethoxzolamide, topiramate, sulpiride, indisulam, celecoxib, and sulthiame were medium potency CAS2 inhibitors (*K*_I_s of 143–857 nM). Many other sulfonamides showed affinities in the high micromolar range or were ineffective as CAS1/2 inhibitors. Small changes in the structure of the inhibitor led to important differences of the activity. As these enzymes may show applications for the removal of anthropically generated polluting gases, finding modulators of their activity may be crucial for designing environmental-friendly CO_2_ capture processes.

## 1. Introduction

*Sordaria macrospora* is a filamentous ascomycete and a model organism for investigating the sexual fruiting body (perithecia) formation, due to the fact that being a homothallic fungus (i.e. self-fertile), it is easily genetically tractable, and well suited for large-scale genomic, transcriptomic, and proteomic studies^1^. The proteins involved in chromatin remodelling and transcriptional regulation of the fruiting body development, as well as the primary and secondary metabolic processes involved in nutrient recycling by autophagy were also understood in greater detail by using S*. macrospora* as a model organism^1^.

Recently, our groups cloned, expressed, and investigated in some detail two β-carbonic anhydrases (CAs, EC 4.2.1.1) encoded in the genome of this fungus, nominated CAS1 and CAS2, which showed a good catalytic activity for the physiologic reaction catalysed by these enzymes, that is, hydration of CO_2_ with formation of bicarbonate and protons^2^. Indeed, CAs belonging to at least two of the seven genetical families known to date, are widespread in fungi^3^^,^^4^, where they are involved in crucial physiologic processes such as, among others, pH regulation and anaplerotic/biosynthetic reactions leading to fatty acids, amino acids, nucleic acids, and other biomolecules^2–5^. Furthermore, both protons and bicarbonate, the reactions products of the enzyme catalysed CO_2_ hydration, are important for chemosensing, a process which regulates fundamental physiologic processes in fungi, such as the type of growth, the production of spores, and for the pathogenic yeasts, also virulence, survival in the host environment, and production of mycotoxins^2–6^. It is thus understandable that CAs^7–10^ have been extensively investigated in the last decade especially in pathogenic fungi, such as *Candida albicans*^11^^,^^12^, *Candida glabrata*^13^^,^^14^, *Cryptococcus neoformans*^15^^,^^16^, *Malassezia globosa*^17^^,^^18^ and to a lower extent in *Saccharomyces cerevisiae*^19^. All these fungi, similar to *S. macrospora* encode for β-CAs, but α-class enzymes were also reported in some species of *Aspergillus*, such as *Aspergillus terreus, Aspergillus oryzae, Aspergillus flavus, Aspergillus niger, Aspergillus fumigatus, Aspergillus nidulans*, and *Aspergillus clavatus*^20^. *S. macrospora* also encodes for an α-CA^20^c. However, the most investigated such organisms from the medicinal chemistry viewpoint, encode for β-CAs. For such enzymes, many inhibition studies are available, in the search of compounds which may interfere with the life cycle of these pathogens^11–19^. In the case of CAS1 and CAS2, only anion inhibitors were investigated, which generally showed low affinity for both isoforms, as expected for this class of CA inhibitors (CAIs)^21^. Thus, in this paper we report the first sulfonamide inhibition study of these enzymes, considering the fact that sulfonamides and their isosteres (sulfamates, sulfamides) are the main class of CAIs, with many such compounds possessing clinical applications for the treatment and prevention of many diseases in which CA activity and expression is dysregulated^22–25^.

## 2. Materials and methods

### 2.1. Chemistry

Sulfonamides **1**–**24** and the clinically used agents **AAZ**–**HCT** were either commercially available, highest purity reagents from Sigma-Aldrich (Milan, Italy), or were reported earlier by one of our groups^22–25^.

### 2.2. CA inhibition assay

An Applied Photophysics stopped-flow instrument has been used for assaying the CA catalysed CO_2_ hydration activity^26^. Phenol red (at a concentration of 0.2 mM) has been used as indicator, working at the absorbance maximum of 557 nm, with 20 mM TRIS (pH 8.3) as buffer, and 20 mM Na_2_SO_4_ (for maintaining constant the ionic strength), following the initial rates of the CA-catalysed CO_2_ hydration reaction for a period of 10–100 s. The CO_2_ concentrations ranged from 1.7 to 17 mM for the determination of the kinetic parameters and inhibition constants. For each inhibitor at least six traces of the initial 5–10% of the reaction have been used for determining the initial velocity. The uncatalysed rates were determined in the same manner and subtracted from the total observed rates. Stock solutions of inhibitor (0.1 mM) were prepared in distilled-deionized water and dilutions up to 0.01 nM were done thereafter with the assay buffer. Inhibitor and enzyme solutions were preincubated together for 15 min at room temperature prior to assay, in order to allow for the formation of the E–I complex. The inhibition constants were obtained by non-linear least-squares methods using PRISM 3 and the Cheng–Prusoff equation, as reported earlier^22–25^, and represent the mean from at least three different determinations. All CA isofoms were recombinant ones obtained in-house as reported earlier^2^^,^^9^.

## 3. Results and discussion

We investigated the susceptibility of CAS1 and CAS2 to inhibition with the main class of CAIs, the sulfonamides and their isosteres (sulfamates/sulfamides)^9^^,^^10^^,^^22–25^. A panel of 40 such derivatives were included in this study. Derivatives **1**–**24** and **AAZ**–**HCT** (Figure 1) are either simple aromatic/heterocyclic sulfonamides widely used as building blocks for obtaining new families of such pharmacological agents^9^^,^^10^^,^^22–25^, or they are clinically used agents, among which acetazolamide **AAZ**, methazolamide **MZA**, ethoxzolamide **EZA**, and dichlorophenamide **DCP**, are the classical, systemically acting antiglaucoma CAIs^9^. Dorzolamide **DZA** and brinzolamide **BRZ** are topically-acting antiglaucoma agents, benzolamide **BZA** is an orphan drug belonging to this class of pharmacological agents, whereas topiramate **TPM**, zonisamide **ZNS**, and sulthiame **SLT** are widely used antiepileptic drugs^9^^,^^22^^,^^25^. Sulpiride **SLP** and indisulam **IND** were also shown by our group to belong to this class of pharmacological agents, together with the COX2 selective inhibitors celecoxib **CLX** and valdecoxib **VLX**^9^. Saccharin and the diuretic hydrochlorothiazide **HCT** are also known to act as CAIs, and were included in this study^22–25^.

Figure 1.Sulfonamides and sulfamates investigated in this article as CAS1/2 inhibitors.

**Figure:**
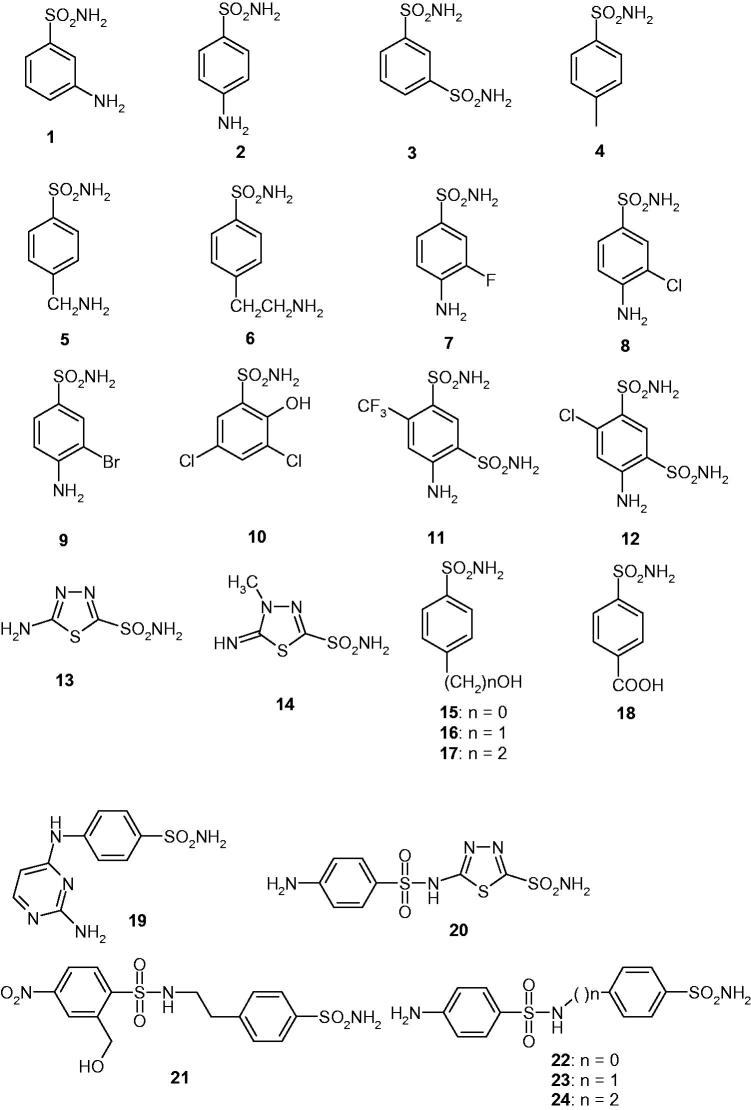

**Figure:**
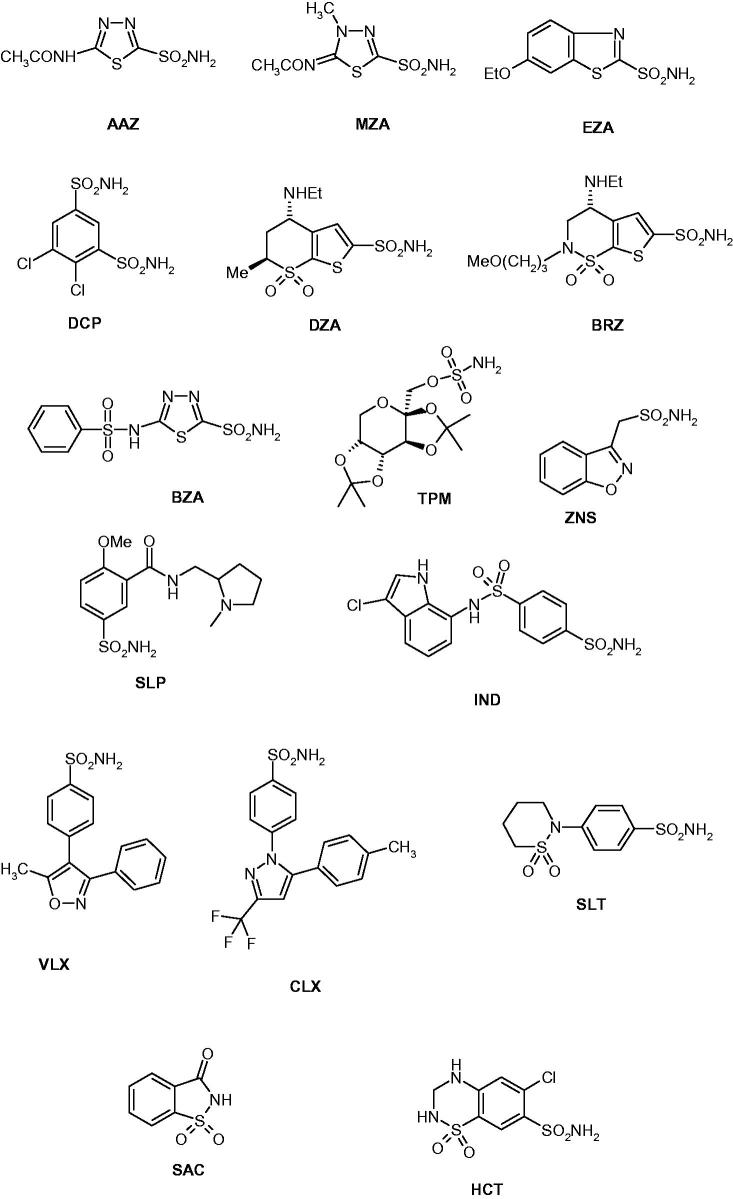

Data of Table 1 show that both CAS1 and CAS2 possess catalytic activity for the hydration of CO_2_ to bicarbonate and protons, with kinetic constants which are lower compared to those of the human (h) isoforms hCA I and II (the last enzyme is one of the best catalysts known in nature)^9^. However, even if these parameters are lower, both enzymes possess a significant catalytic activity, with *k*_cat_/*K*_m_ values >10^6^ s^−1^. Furthermore, this activity is inhibited by the clinically sued sulfonamide acetazolamide, a standard CAI, as shown in Table 1, although with inhibition constants in the high nanomolar range (*K*_I_ of 445 nM for CAS1 and of 816 nM for CAS2)^2^.

**Table 1.** Kinetic parameters for the CO_2_ hydration reaction catalysed by the human cytosolic isozymes hCA I and II (α-class CAs) at 20 °C and pH 7.5 in 10 mM HEPES buffer and 20 mM NaClO_4_, and the β-CAs CAS1 and CAS2 of *Sordaria macrospora* measured at 20 °C, pH 8.3 in 20 mM TRIS buffer and 20 mM NaClO_4_.

Isozyme	Activity level	*k*_cat_	*k*_cat_/*K*_m_ (s^−1^)	*K*_I_ (acetazolamide) (nM)
hCA I	Moderate	2.0 × 10^5^	5.0 × 10^7^	250
hCA II	Very high	1.4 × 10^6^	1.5 × 10^8^	12
CAS1	Low	1.2 × 10^4^	1.30 × 10^6^	445
CAS2	Low	1.3 × 10^4^	1.21 ×	816

Inhibition data with the clinically used sulfonamide acetazolamide (5-acetamido-1,3,4-thiadiazole-2-sulfonamide) are also provided.

Inhibition data of CAS1 and CAS2 with the sulfonamides shown in Figure 1 (compounds **1**–**24** and **AAZ**–**HCT**) are presented in Table 2, in which the hCA I/II inhibition with the same set of derivatives is also shown for comparison reasons.

**Table 2.** Inhibition of human isoforms hCA I and hCA II, and of the β-class fungal enzymes CAS1 and CAS2 with sulfonamides **1**–**24** and the clinically used drugs **AAZ**–**HCT**.

Inhibitor/enzyme class	*K*_I_^a^ (nM)			
	hCA I^b^	hCA II^b^	CAS1^c^	CAS2^c^
	α	α	β	β
1	28,000	300	361	386
2	25,000	240	144	3480
3	79^c^	8	225	3630
4	78,500	320	47.1	6900
5	25,000	170	323	8720
6	21,000	160	241	7650
7	8300	60	43.2	7360
8	9800	110	79.6	9120
9	6500	40	580	12,000
10	7300	54	>50,000	23,500
11	5800	63	890	18,700
12	8400	75	3350	>50,000
13	8600	60	8650	48.1
14	9300	19	7215	280
15	5500	80	3160	143
16	9500	94	4520	92.5
17	21,000	125	>50,000	390
18	164	46	4435	3250
19	109	33	475	6760
20	6	2	363	9880
21	69	11	4550	4060
22	164	46	1985	25,200
23	109	33	282	>50,000
24	95	30	294	>50,000
AAZ	250	12	445	816
MZA	50	14	421	8140
EZA	25	8	440	3170
DCP	1200	38	1220	5790
DZA	50,000	9	360	742
BRZ	45,000	3	451	739
BZA	15	9	2115	410
TPM	250	10	414	673
ZNS	56	35	1820	1885
SLP	1200	40	1715	670
IND	31	15	4240	216
VLX	54,000	43	4425	3730
CLX	50,000	21	2513	857
SLT	374	9	3210	496
SAC	18,540	5959	5280	7075
HCT	328	290	3350	6680

a Errors in the range of 5–10% of the reported data, from three different assays (data not shown).

b Human recombinant isozymes, stopped flow CO_2_ hydrase assay method, from Refs. [9,22–25].

c Recombinant fungal enzyme, stopped flow CO_2_ hydrase assay method, this work.

The following structure–activity relationship (SAR) can be observed from the data of Table 2:

- Several sulfonamides were ineffective as CAS1/2 inhibitors, with *K*_I_s > 50 µM. They include **10** and **17** for CAS1, and **12**, **23**, and **24** for CAS2 (Table 2).
- For CAS1, a range of derivatives, among which **12**–**16**, **18**, **21**, **22**, **DCP**, **BRZ**, and **ZNS**–**HCT**, showed weak inhibitory action, with inhibition constants in the micromolar range, more precisely of 1.22–8.65 µM. They include a large variety of different chemotypes, such as the aromatic 1,3-benzene-disulfonamide **12**, the heterocyclic precursors of two clinically used agents (**AAZ**, **MZA**) **13** and **14**, as well as the elongated molecule sulfonamide of the sulfonylated-aminosulfonamide type **21** and **22**, in addition to the clinically used agents which possess an even higher diversity of scaffolds. It is thus impossible to draw detailed SAR conclusions based on these very variable chemotypes with this modest activity.
- A large number of derivatives behaved as medium potency CAS1 inhibitors, with *K*_I_s in the range of 144–890 nM (Table 1). They include **1**–**3**, **5**, **6**, **9**, **11**, **19**, **20**, **23**, **24**, **AAZ**, **MZA**, **EZA**, **DZA**, **BRZ**, and **TPM**. All of them belong to the sulfonamide class, except **TPM** which is the only sulfamate investigated here. From the chemical viewpoint, they also possess a rather high variability, but some of these chemotypes are easier to rationalize. Thus, 3- or 4-substituted benzenesulfonamides with compact moieties (amino, sulfamoyl, aminoalkyl), such as in derivatives **1**–**3**, **5**, and **6**, lead to a rather effective CAS inhibitory action compared to the bulkier derivatives discussed above (e.g. **11**, **12**, **21**, **22**, etc.). The halogenosubstituted sulfanilamides show a good activity (especially for light halogens incorporating derivatives which will be discussed shortly), with the bromosubstituted derivative **9** being less effective than sulfanilamide **2**, whereas the fluoro- and chloro-containing derivatives **7** and **8** being much better CAS1 inhibitors than the lead **2**. It is also interesting to note the difference between the two 1,3-benzene-disulfonamides **11** and **12**, with the trifluoromethyl derivative **11** being 3.76 times a better inhibitor compared to the structurally related chlorine derivative **12**. Compounds **20**, **23**, and **24** belong to the sulfonylated-aminosulfonamide class of CAIs, as **21** and **22** discussed earlier, but in the case of **20** the presence of the 1,3,4-thiadiazole-2-sulfonamide head probably leads to the enhanced inhibitory effect, whereas for **23** and **24**, the longer spacers between the two parts of the molecule (compared to the spacer from **20**, which is in fact absent) produce the same effect. Thus, in these cases the SAR is rather well defined, demonstrating that small structural changes in the molecule of the inhibitor lead to drastic changes in the affinity for the enzyme. Among the clinically used sulfonamides/sulfamates, **AAZ**, **MZA**, **EZA**, **DZA**, **BRZ**, and **TPM** are in this category of medium potency inhibitors. It should be noted that whereas **AAZ** and **MZA** possess rather compact, monocyclic scaffolds, the ones from **EZA**, **DZA**, and **BRZ** are much bulkier, which proves that the active site of the enzyme may accommodate even these sterically hindered sulfonamides. The same situation was observed for the even bulkier sugar sulfamate **TPM**, which has an activity quite similar to that of **MZA** (Table 2).
- The best CAS1 inhibitors were tosylamide **4**, 3-fluorosulfanilamide **7**, and 3-chlorosulfanilamide **8**, which had *K*_I_s in the range of 43.2–79.6 nM. Thus, these compounds were one order of magnitude more effective as CAS1 inhibitors compared to the clinically used agents mentioned above (**AAZ**, **MZA**, **TPM**, etc.). The increase in the inhibition power of **7** and **8** over sulfanilamide **2** was in the range of 1.80–3.33-fold, demonstrating that it may be possible to obtain highly effective and probably isoform-specific CAS1 inhibitors through a drug design program, using these derivatives as lead molecules.
- CAS2 was poorly inhibited by **9**–**11** and **22**, with *K*_I_s in the range of 12.0–25.2 µM. These derivatives incorporate two or three substituents on the benzenesulfonamide scaffold (as in **9**–**11**) or have the elongated sulfonylated-aminosulfonamide scaffold (**22**). Another rather large series of derivatives showed slightly better but still micromolar affinity for CAS2. They include **2**–**8**, **18**–**21**, **MZA**, **EZA**, **DCP**, **ZNS**, **VLX**, **SAC**, and **HCT**, and their inhibition constants range between 1.88 and 9.88 µM (Table 2). As discussed above, these inhibitors belong to heterogeneous chemical classes, such as the mono- or poly-substituted benzenesulfonamides (**2**–**8**, **18**, **DCP**), the derivatives with bulkier scaffolds (**19**–**21**, **EZA**, **VLX**, **HCT**) but other derivatives such as saccharin **SAC** or zonisamide (**ZNS**) which possess rather unique structural features among the library of investigated compounds.
- Medium potency CAS2 inhibitors were **1**, **14**, **15**, **17**, **AAZ**, **DZA**–**TPM**, **SLP**, **IND**, **CLX**, and **SLT**, with *K*_I_s in the range of 143–857 nM (Table 2). Again small structural changes in the molecule of the inhibitor lead to important changes of activity. For example, in the isomeric pair **1** and **2**, the amino moiety in *meta* (as in **1**) leads to a nine times better CAS2 inhibitory power compared to the *para*-amino substituted derivative **2**. Comparing the *p*-amino- and *p*-hydroxy-benzenesulfonamides **2** and **15**, the latter one is 24.33 times a better CAS2 inhibitor compared to sulfanilamide **2**, showing again that quite similar compounds from the structural viewpoint may interact in a very diverse manner with the enzyme. Another striking example is constituted by the deacetylated acetazolamide precursor **13** (the best CAS2 inhibitor detected here), which is almost 17 times a better inhibitor compared to **AAZ**.
- The best CAS2 inhibitors detected here were 5-amino-1,3,4-thiadiazole-2-sulfonamide (the deacetylated acetazolamide precursor **13**) and 4-hydroxymethyl-benzenesulfonamide **16**, which showed *K*_I_s in the range of 48.1–92.5 nM.
- The inhibition profiles with sulfonamides and one sulfamate of CAS1 and CAS2 were very different between the two fungal isoforms, and also when compared to the inhibition of the human, α-class enzymes hCA I and II; for which many of the investigated derivatives acted with efficiencies in the low nanomolar range (Table 2). This is in fact to be expected, considering that the fungal and the human isoforms belong to two distinct genetic families.

## 4. Conclusions

We report the first sulfonamide inhibition study of two fungal β-CAs from *S. macrospora*, CAS1 and CAS2. CAS1 was efficiently inhibited by tosylamide, 3-fluorosulfanilamide and 3-chlorosulfanilamide (*K*_I_s in the range of 43.2–79.6 nM), whereas acetazolamide, methazolamide, topiramate, ethoxzolamide, dorzolamide, and brinzolamide were medium potency inhibitors (*K*_I_s in the range of 360–445 nM). CAS2 was less sensitive to sulfonamide inhibitors. The best CAS2 inhibitors were 5-amino-1,3,4-thiadiazole-2-sulfonamide (the deacetylated acetazolamide precursor) and 4-hydroxymethyl-benzenesulfonamide, with *K*_I_s in the range of 48.1–92.5 nM. Acetazolamide, dorzolamide, ethoxzolamide, topiramate, sulpiride, indisulam, celecoxib, and sulthiame were medium potency CAS2 inhibitors (*K*_I_s of 143–857 nM). Many other sulfonamides showed affinities in the high micromolar range or were ineffective as CAS1/2 inhibitors. Small changes in the structure of the inhibitor led to important differences of activity. As these enzymes may show applications for the removal of anthropically generated polluting gases, finding modulators of their activity may be crucial for designing environmental-friendly CO_2_ capture processes.

## References

[1] (a) Zickler D, Espagne E. Sordaria, a model system to uncover links between meiotic pairing and recombination. Semin Cell Dev Biol 2016;54:149–57. (b) Teichert I, Nowrousian M, Pöggeler S, Kück U. The filamentous fungus *Sordaria macrospora* as a genetic model to study fruiting body development. Adv Genet 2014;87:199–244. 10.1016/j.semcdb.2016.02.012 PMC5510351 26877138

[2] Lehneck R, Neumann P, Vullo D, et al Crystal structures of two tetrameric β-carbonic anhydrases from the filamentous ascomycete *Sordaria macrospora*. FEBS J 2014;281:1759–72. 24506675 10.1111/febs.12738

[3] (a) Elleuche S, Pöggeler S Evolution of carbonic anhydrases in fungi. Curr Genet 2009;55:211–22. (b) Elleuche S, Pöggeler S Carbonic anhydrases in fungi. Microbiology 2010;1:23–9. (c) Lehneck R, Pöggeler S Fungal carbonic anhydrases and their inhibition. Top Med Chem 2017;22:95–110. (d) Lehneck R, Pöggeler S. A matter of structure: structural comparison of fungal carbonic anhydrases. Appl Microbiol Biotechnol 2014;98:8433–41.

[4] (a) Hall RA, De Sordi L, MacCallum DM, et al CO_2_ acts as a signalling molecule in populations of the fungal pathogen *Candida albicans*. PLoS Pathog 2010;6:e1001193 (b) Cottier F, Leewattanapasuk W, Kemp LR, et al Carbonic anhydrase regulation and CO2 sensing in the fungal pathogen *Candida glabrata* involves a novel Rca1p ortholog. Bioorg Med Chem 2013;2:1549–54. (c) Mogensen EG, Janbon G, Chaloupka J, et al *Cryptococcus neoformans* senses CO_2_ through the carbonic anhydrase Can2 and the adenylyl cyclase Cac1. Eukaryot Cell 2006;5:103–11.

[5] (a) Elleuche S, Pöggeler S Beta-carbonic anhydrases play a role in fruiting body development and ascospore germination in the filamentous fungus *Sordaria macrospora*. PLoS One 2009;4:e5177 (b) Aguilera J, Van Dijken JP, De Winde JH, Pronk JT. Carbonic anhydrase (Nce103p): an essential biosynthetic enzyme for growth of *Saccharomyces cerevisiae* at atmospheric carbon dioxide pressure. Biochem J 2005;391:311–16. 10.1371/journal.pone.0005177 PMC2664464 19365544

[6] Li YP, Tang X, Wu W, et al The ctnG gene encodes carbonic anhydrase involved in mycotoxin citrinin biosynthesis from *Monascus aurantiacus*. Food Addit Contam Part A Chem Anal Control Expo Risk Assess 2015;32:577–83. 25482072 10.1080/19440049.2014.990993

[7] (a) Supuran CT Structure and function of carbonic anhydrases. Biochem J 2016;4:2023–32. (b) Supuran CT. How many carbonic anhydrase inhibition mechanisms exist? J Enzyme Inhib Med Chem 2016;31:345–60. (c) Supuran CT. Carbonic anhydrases: novel therapeutic applications for inhibitors and activators. Nat Rev Drug Discov 2008;7:168–81.

[8] (a) Supuran CT. Carbonic anhydrases: from biomedical applications of the inhibitors and activators to biotechnological use for CO_2_ capture. J Enzyme Inhib Med Chem 2013;28:229–30. (b) Fabrizi F, Mincione F, Somma T, et al A new approach to antiglaucoma drugs: carbonic anhydrase inhibitors with or without NO donating moieties. Mechanism of action and preliminary pharmacology. J Enzyme Inhib Med Chem 2012;27:138–47. (c) Capasso C, Supuran CT. Bacterial, fungal and protozoan carbonic anhydrases as drug targets. Expert Opin Ther Targets 2015;19:1689–704.

[9] (a) Supuran CT. Structure-based drug discovery of carbonic anhydrase inhibitors. J Enzyme Inhib Med Chem 2012;27:759–72. (b) Scozzafava A, Menabuoni L, Mincione F, Supuran CT. Carbonic anhydrase inhibitors. A general approach for the preparation of water-soluble sulfonamides incorporating polyamino-polycarboxylate tails and of their metal complexes possessing long-lasting, topical intraocular pressure-lowering properties. J Med Chem 2002;45:1466–76. (c) Alterio VD, Fiore A, D’Ambrosio K, et al Multiple binding modes of inhibitors to carbonic anhydrases: how to design specific drugs targeting 15 different isoforms? Chem Rev 2012;112:4421–68.

[10] (a) Masini E, Carta F, Scozzafava A, Supuran CT. Antiglaucoma carbonic anhydrase inhibitors: a patent review. Expert Opin Ther Pat 2013;23:705–16. (b) Puccetti L, Fasolis G, Vullo D, et al Carbonic anhydrase inhibitors. Inhibition of cytosolic/tumor-associated carbonic anhydrase isozymes I, II, IX, and XII with Schiff's bases incorporating chromone and aromatic sulfonamide moieties, and their zinc complexes. Bioorg Med Chem Lett 2005;15:3096–101. (c) Scozzafava A, Menabuoni L, Mincione F, et al Carbonic anhydrase inhibitors: perfluoroalkyl/aryl-substituted derivatives of aromatic/heterocyclic sulfonamides as topical intraocular pressure-lowering agents with prolonged duration of action. J Med Chem 2000;43:4542–51. (d) Supuran CT, Mincione F, Scozzafava A, et al Carbonic anhydrase inhibitors—part 52. Metal complexes of heterocyclic sulfonamides: a new class of strong topical intraocular pressure-lowering agents in rabbits. Eur J Med Chem 1998;33:247–54.

[11] (a) Innocenti A, Mühlschlegel FA, Hall RA, et al Carbonic anhydrase inhibitors: inhibition of the beta-class enzymes from the fungal pathogens *Candida albicans* and *Cryptococcus neoformans* with simple anions. Bioorg Med Chem Lett 2008;18:5066–70. (b) Innocenti A, Hall RA, Schlicker C, et al Carbonic anhydrase inhibitors. Inhibition of the beta-class enzymes from the fungal pathogens *Candida albicans* and *Cryptococcus neoformans* with aliphatic and aromatic carboxylates. Bioorg Med Chem 2009;17:2654–7. 10.1016/j.bmcl.2008.07.122 18723348

[12] (a) Innocenti A, Hall RA, Schlicker C, et al Carbonic anhydrase inhibitors. Inhibition and homology modeling studies of the fungal beta-carbonic anhydrase from *Candida albicans* with sulfonamides. Bioorg Med Chem 2009;1:4503–9. (b) Carta F, Innocenti A, Hall RA, et al Carbonic anhydrase inhibitors. Inhibition of the β-class enzymes from the fungal pathogens *Candida albicans* and *Cryptococcus neoformans* with branched aliphatic/aromatic carboxylates and their derivatives. Bioorg Med Chem Lett 2011;2:2521–6. (c) Rami M, Innocenti A, Montero JL, et al Synthesis of rhodamine B-benzenesulfonamide conjugates and their inhibitory activity against human α- and bacterial/fungal β-carbonic anhydrases. Bioorg Med Chem Lett 2011;21:5210–13.

[13] (a) Innocenti A, Leewattanapasuk W, Mühlschlegel FA, et al Carbonic anhydrase inhibitors. Inhibition of the beta-class enzyme from the pathogenic yeast *Candida glabrata* with anions. Bioorg Med Chem Lett 2009;1:4802–5. (b) Monti SM, Maresca A, Viparelli F, et al Dithiocarbamates are strong inhibitors of the beta-class fungal carbonic anhydrases from *Cryptococcus neoformans, Candida albicans* and *Candida glabrata*. Bioorg Med Chem Lett 2012;22:859–62. (c) Vullo D, Leewattanapasuk W, Mühlschlegel FA, et al Carbonic anhydrase inhibitors: inhibition of the β-class enzyme from the pathogenic yeast *Candida glabrata* with sulfonamides, sulfamates and sulfamides. Bioorg Med Chem Lett 2013;23:2647–52.

[14] (a) Innocenti A, Leewattanapasuk W, Manole G, et al Carbonic anhydrase activators: activation of the beta-carbonic anhydrase from the pathogenic yeast *Candida glabrata* with amines and amino acids. Bioorg Med Chem Lett 2010;2:1701–4. (b) Supuran CT. Bortezomib inhibits bacterial and fungal β-carbonic anhydrases. Bioorg Med Chem 2016;24:4406–9. 10.1016/j.bmcl.2010.01.054 20129782

[15] (a) Schlicker C, Hall RA, Vullo D, et al Structure and inhibition of the CO_2_-sensing carbonic anhydrase Can2 from the pathogenic fungus *Cryptococcus neoformans*. J Mol Biol 2009;385:1207–20. (b) Innocenti A, Winum JY, Hall RA, et al Carbonic anhydrase inhibitors. Inhibition of the fungal beta-carbonic anhydrases from *Candida albicans* and *Cryptococcus neoformans* with boronic acids. Bioorg Med Chem Lett 2009;19:2642–5. (c) Davis RA, Hofmann A, Osman A, et al Natural product-based phenols as novel probes for mycobacterial and fungal carbonic anhydrases. J Med Chem 2011;54:1682–92. (c) Akdemir A, Güzel-Akdemir Ö, Karalı N, Supuran CT. Isatin analogs as novel inhibitors of *Candida* spp. β-carbonic anhydrase enzymes. Bioorg Med Chem 2016;24:1648–52.

[16] (a) Innocenti A, Hall RA, Scozzafava A, et al Carbonic anhydrase activators: activation of the beta-carbonic anhydrases from the pathogenic fungi *Candida albicans* and *Cryptococcus neoformans* with amines and amino acids. Bioorg Med Chem 2010;1:1034–7. (b) Güzel O, Maresca A, Hall RA, et al Carbonic anhydrase inhibitors. The beta-carbonic anhydrases from the fungal pathogens *Cryptococcus neoformans* and *Candida albicans* are strongly inhibited by substituted-phenyl-1H-indole-5-sulfonamides. Bioorg Med Chem Lett 2010;2:2508–11. (c) Nocentini A, Cadoni R, Del Prete S, et al Benzoxaboroles as efficient inhibitors of the β-carbonic anhydrases from pathogenicf: activity and modeling study. ACS Med Chem Lett 2017;8:1194–8.

[17] (a) Hewitson KS, Vullo D, Scozzafava A, et al Molecular cloning, characterization, and inhibition studies of a β-carbonic anhydrase from *Malassezia globosa*, a potential antidandruff target. J Med Chem 2012;55:3513–20. (b) Del Prete S, Vullo D, Osman SM, et al Anion inhibition studies of the dandruff-producing fungus *Malassezia globosa* β-carbonic anhydrase MgCA. Bioorg Med Chem Lett 2015;25:5194–8. (c) Vullo D, Del Prete S, Nocentini A, et al Dithiocarbamates effectively inhibit the β-carbonic anhydrase from the dandruff-producing fungus *Malassezia globosa*. Bioorg Med Chem 2017;25:1260–5. (d) Nocentini A, Vullo D, Del Prete S, et al Inhibition of the β-carbonic anhydrase from the dandruff-producing fungus *Malassezia globosa* with monothiocarbamates. J Enzyme Inhib Med Chem 2017;32:1064–70.

[18] (a) Singh S, Supuran CT. In silico modeling of β-carbonic anhydrase inhibitors from the fungus *Malassezia globosa* as antidandruff agents. J Enzyme Inhib Med Chem 2016;31:417–24. (b) Del Prete S, De Luca V, Vullo D, et al A new procedure for the cloning, expression and purification of the β-carbonic anhydrase from the pathogenic yeast *Malassezia globosa*, an anti-dandruff drug target. J Enzyme Inhib Med Chem 2016;31:1156–61. (c) Vullo D, Del Prete S, Capasso C, Supuran CT. Carbonic anhydrase activators: activation of the β-carbonic anhydrase from *Malassezia globosa* with amines and amino acids. Bioorg Med Chem Lett 2016;26:1381–5. (d) Entezari Heravi Y, Bua S, Nocentini A, et al Inhibition of *Malassezia globosa* carbonic anhydrase with phenols. Bioorg Med Chem 2017;25:2577–82. (e) Angiolella L, Carradori S, Maccallini C, et al Targeting *Malassezia* species for novel synthetic and natural antidandruff agents. Curr Med Chem 2017;24:2392–412.

[19] (a) Isik S, Kockar F, Arslan O, et al Carbonic anhydrase inhibitors. Inhibition of the beta-class enzyme from the yeast *Saccharomyces cerevisiae* with anions. Bioorg Med Chem Lett 2008;1:6327–231. (b) Isik S, Kockar F, Aydin M, et al Carbonic anhydrase inhibitors: inhibition of the beta-class enzyme from the yeast *Saccharomyces cerevisiae* with sulfonamides and sulfamates. Bioorg Med Chem 2009;1:1158–63. (c) Isik S, Kockar F, Aydin M, et al. Carbonic anhydrase activators: activation of the beta-carbonic anhydrase Nce103 from the yeast *Saccharomyces cerevisiae* with amines and amino acids. Bioorg Med Chem Lett 2009;1:1662–5. (d) Isik S, Guler OO, Kockar F, et al *Saccharomyces cerevisiae* β-carbonic anhydrase: inhibition and activation studies. Curr Pharm Des 2010;1:3327–36. (e) Bozdag M, Carta F, Vullo D, et al Dithiocarbamates with potent inhibitory activity against the *Saccharomyces cerevisiae* β-carbonic anhydrase. J Enzyme Inhib Med Chem 2016;3:132–6. (f) Bilginer S, Unluer E, Gul HI, et al Carbonic anhydrase inhibitors. Phenols incorporating 2- or 3-pyridyl-ethenylcarbonyl and tertiary amine moieties strongly inhibit *Saccharomyces cerevisiae* β-carbonic anhydrase. J Enzyme Inhib Med Chem 2014;29:495–9.

[20] (a) Cuesta-Seijo JA, Borchert MS, Navarro-Poulsen JC, et al Structure of a dimeric fungal α-type carbonic anhydrase. FEBS Lett 2011;5:1042–8. (b) Tobal JM, Balieiro ME. Role of carbonic anhydrases in pathogenic micro-organisms: a focus on *Aspergillus fumigatus*. J Med Microbiol 2014;6:15–27. (c) Lehenck R, Elleche S, Pöggeler S. The filamentous ascomycete *Sordaria macrospora* can survive in ambient air without carbonic anhydrases. Mol Microbiol 2014;92:931–44.

[21] De Simone G, Supuran CT. (In)organic anions as carbonic anhydrase inhibitors. J Inorg Biochem 2012;111:117–29. 22192857 10.1016/j.jinorgbio.2011.11.017

[22] Carta F, Scozzafava A, Supuran CT Sulfonamides: a patent review (2008–2012). Expert Opin Ther Pat 2012;22:747–58. 22697257 10.1517/13543776.2012.698264

[23] (a) Supuran CT, Vullo D, Manole G, et al Designing of novel carbonic anhydrase inhibitors and activators. Curr Med Chem Cardiovasc Hematol Agents 2004;2:49–68. (b) De Simone G, Langella E, Esposito D, et al Insights into the binding mode of sulphamates and sulphamides to hCA II: crystallographic studies and binding free energy calculations. J Enzyme Inhib Med Chem 2017;32:1002–11. 15328829

[24] (a) Carta F, Supuran CT. Diuretics with carbonic anhydrase inhibitory action: a patent and literature review (2005–2013). Expert Opin Ther Pat 2013;23:681–91. (b) Scozzafava A, Supuran CT, Carta F. Antiobesity carbonic anhydrase inhibitors: a literature and patent review. Expert Opin Ther Pat 2013;23:725–35. (c) Abbate F, Winum JY, Potter BV, et al Carbonic anhydrase inhibitors: X-ray crystallographic structure of the adduct of human isozyme II with EMATE, a dual inhibitor of carbonic anhydrases and steroid sulfatase. Bioorg Med Chem Lett 2004;14:231–4.

[25] (a) Supuran CT. Carbonic anhydrase inhibition and the management of neuropathic pain. Expert Rev Neurother 2016;16:961–8. (b) Di Cesare Mannelli L, Micheli L, Carta F, et al Carbonic anhydrase inhibition for the management of cerebral ischemia: in vivo evaluation of sulfonamide and coumarin inhibitors. J Enzyme Inhib Med Chem 2016;31:894–9. (c) Margheri F, Ceruso M, Carta F, et al Overexpression of the transmembrane carbonic anhydrase isoforms IX and XII in the inflamed synovium. J Enzyme Inhib Med Chem 2016;31:60–3.

[26] Khalifah RG. The carbon dioxide hydration activity of carbonic anhydrase. I. Stop-flow kinetic studies on the native human isoenzymes B and C. J Biol Chem 1971;246:2561–73. 4994926

